# The Human SLC1A5 Neutral Amino Acid Transporter Catalyzes a pH-Dependent Glutamate/Glutamine Antiport, as Well

**DOI:** 10.3389/fcell.2020.00603

**Published:** 2020-07-08

**Authors:** Mariafrancesca Scalise, Tiziano Mazza, Gilda Pappacoda, Lorena Pochini, Jessica Cosco, Filomena Rovella, Cesare Indiveri

**Affiliations:** ^1^Department DiBEST (Biologia, Ecologia, Scienze della Terra), Unit of Biochemistry and Molecular Biotechnology, University of Calabria, Arcavacata, Italy; ^2^CNR Institute of Biomembranes, Bioenergetics and Molecular Biotechnologies (IBIOM), Bari, Italy

**Keywords:** amino acid, SLC, glutamine, glutamate, membrane, transport, proteoliposome

## Abstract

ASCT2 is a neutral amino acid transporter, which catalyzes a sodium-dependent obligatory antiport among glutamine and other neutral amino acids. The human ASCT2 over-expressed in *Pichia pastoris* and reconstituted in proteoliposomes has been employed for identifying alternative substrates of the transporter. The experimental data highlighted that hASCT2 also catalyzes a sodium-dependent antiport of glutamate with glutamine. This unconventional antiport shows a preferred sidedness: glutamate is inwardly transported in exchange for glutamine transported in the counter direction. The orientation of the transport protein in proteoliposomes is the same as in the cell membrane; then, the observed sidedness corresponds to the transport of glutamate from the extracellular to the intracellular compartment. The competitive inhibition exerted by glutamate on the glutamine transport together with the docking analysis indicates that the glutamate binding site is the same as that of glutamine. The affinity for glutamate is lower than that for neutral amino acids, while the transport rate is comparable to that measured for the asparagine/glutamine antiport. Differently from the neutral amino acid antiport that is insensitive to pH, the glutamate/glutamine antiport is pH-dependent with optimal activity at acidic pH on the external (extracellular) side. The stimulation of glutamate transport by a pH gradient suggests the occurrence of a proton flux coupled to the glutamate transport. The proton transport has been detected by a spectrofluorometric method. The rate of proton transport correlates well with the rate of glutamate transport indicating a 1:1 stoichiometry H^+^: glutamate. The glutamate/glutamine antiport is also active in intact HeLa cells. On a physiological point of view, the described antiport could have relevance in some districts in which a glutamate/glutamine cycling is necessary, such as in placenta.

## Introduction

The fifth member of the SLC1 family, ASCT2 (SLC1A5), attracted the attention of the membrane transport scientific community in the last years for its link with the metabolic rewiring occurring in cancer cells. The increased efforts in studying this transporter revealed novel physiological roles in the regulation of the amino acid homeostasis (Scalise et al., 2018a; Freidman et al., 2020) and brought to the resolution of the 3D structure (Garaeva et al., 2018). Since its first isolation in mice and human cells, ASCT2 was described as a sodium-dependent obligatory antiporter of neutral amino acids with specificity toward Ala, Ser, and Cys as indicated by the acronym ASCT2. Soon after its basic functional characterization, glutamine revealed to be the preferred substrate of the protein. This data was obtained in studies conducted in intact cell systems as well as in proteoliposomes using the murine and the human isoforms of ASCT2 either in native form or obtained by over-production in *P. pastoris* (Utsunomiya-Tate et al., 1996; Torres-Zamorano et al., 1998; Oppedisano et al., 2007; Pingitore et al., 2013). Interestingly, the availability of the recombinant hASCT2 together with its reconstitution in proteoliposomes allowed solving some controversies around this protein. As an example, it was demonstrated that the antiport of neutral amino acids, coupled to the movement of at least one sodium ion, is electrogenic in contrast with the previous believing describing an electroneutral exchange of amino acids and sodium. The intracellular sodium is an allosteric regulator of the hASCT2 transport function (Scalise et al., 2014). The kinetics of transport reaction obeys to a random simultaneous mechanism in which the three substrates do not influence the affinity of the transporter toward each other. A trimeric assembly of hASCT2 was proposed by cross-linking experiments. In this quaternary structure, the monomers work independently from each other (Scalise et al., 2014). Later on, the 3D structure of ASCT2 was solved by CryoEM employing the protein over-produced in *P. pastoris* (Pingitore et al., 2013; Garaeva et al., 2018, 2019), confirmed that the protein is organized as a trimer in the plasma membrane. The high affinity of hASCT2 toward glutamine underlies its role in cancer (Bhutia et al., 2015; Scalise et al., 2018a). Indeed, cancer cells are glutamine addicted and require a great supply of this amino acid to sustain their high proliferation rate both in terms of biomass and energy production. Interestingly, ASCT2 is overexpressed in virtually all human cancers, thus, it is not a surprise that this protein became a hot target for drug design (Bhutia et al., 2015; Scalise et al., 2018a). Therefore, one of the most attractive topics around hASCT2 is defining the molecular determinants for the substrate specificity. Understanding this basic aspect is, indeed, fundamental in either physiological studies and pharmacological applications. In this respect, it was recently described that cysteine is not a substrate of ASCT2 but acts as an allosteric regulator driving a glutamine efflux in intact cells as well as in proteoliposomes (Scalise et al., 2015). Then, data on the substrate-binding site were obtained by site-directed mutagenesis (Scalise et al., 2018a), which correlated well with the 3D structure (Garaeva et al., 2018, 2019). Soon after, the modulation of the transport function by cholesterol was described by structure/function relationship studies (Scalise et al., 2019). Again, this last finding correlated well with structural data obtained by CryoEM (Yu et al., 2019). In the frame of substrate specificity, previous results showed that some transporters of the SLC1 family, as well as their bacterial homologs, can be forced to switch the specificity from neutral amino acids to acidic ones (glutamate or aspartate) or vice versa, by mutating some specific residues (Scopelliti et al., 2013, 2018; Canul-Tec et al., 2017). Noteworthy, besides these artificial mutations, the very first report on mice ASCT2 showed Na^+^ and pH-dependent transport of glutamate with a Km in the millimolar range, indicating a lower affinity compared to that of neutral amino acids (Utsunomiya-Tate et al., 1996; Broer et al., 1999). Then, it was shown that glutamate triggered the glutamine efflux in rat astrocytes but without a definitive molecular explanation (Deitmer and Rose, 1996; Broer and Brookes, 2001). The rat ASCT2 reconstituted in liposomes catalyzed a glutamine/glutamate antiport even though at a lower efficiency in comparison to that of neutral amino acids (Oppedisano et al., 2007) correlating with the data collected in intact cells. No data on direct glutamate transport by the human isoform of ASCT2 was available so far, then, we have further dealt with the issue of substrate “adaptation” by investigating the capacity of the hASCT2 to transport glutamate and aspartate. Indeed, we here demonstrated that the wild type hASCT2 can mediate a Na^+^ dependent aspartate_ex_-glutamate_ex_/glutamine_in_ antiport without any artificial modification of its primary structure. This novel aspect represents a step forward in the understanding of the actual physiological role of hASCT2 with potential outcomes also in pharmaceutical applications.

## Materials and Methods

### Materials

The *P. pastoris* wild type strain (X-33), the pPICZB vector, zeocin, Ni-NTA agarose resin were from Invitrogen; PD-10 columns were from GE Healthcare; L-[^3^H]Glutamine and L-[^3^H]glutamic acid were from Perkin Elmer; C_12_E_8_ was from TCI Europe; Cholesterol, Amberlite XAD-4, egg yolk phospholipids (3-sn-phosphatidylcholine from egg yolk), Sephadex G-75, L-glutamine, L-glutamic acid monosodium salt, DEPC, valinomycin, nigericin, pyranine (8-Hydroxypyrene-1,3,6-trisulfonic acid trisodium salt) and all the other reagents were from Sigma-Aldrich.

### Recombinant Production of hASCT2-6His

To produce the recombinant hASCT2-6His protein a previously pointed out approach was employed (Pingitore et al., 2013). In brief: 10 μg of pPICZB-ASCT2-6His WT construct was linearized with *Pme*I. The linearized plasmid was used to transform *P. pastoris* wild type strain X-33 by electroporation (Oberg et al., 2011). Prior large scale protein production, transformed *P. pastoris* cells were selected using YPDS plates containing 2,000 μg/ml zeocin; then, cells were inoculated in BMGY medium (Buffered glycerol-complex medium) and grown at 30°C under rotatory stirring (Scalise et al., 2018b). Then, the BMGY medium was removed by centrifuging *P. pastoris* cells which were resuspended at final OD of 1 in 250 ml BMMY medium (Buffered complex methanol medium) containing 0.5% methanol. The cells were placed in a 2 L conical flask and grown in the same medium at 30°C under rotatory stirring, for 3 days. Fresh methanol was added every 24 h. The *P. pastoris* membrane fraction was prepared using, as starting material, 30-40 g of cells resuspended in 300 ml of a buffer composed by 50 mM Tris–HCl pH 7.4, 150 mM NaCl, 2 mM β-mercaptoethanol and 0.5 mM PMSF. The cell suspension was loaded in the chamber of a bead beater (BioSpec Product) for disruption using glass beads (0.5 mm) for 5 min cycle reaching 90% of cell disruption. Then, the broken cell suspension was centrifuged in a JA10 rotor at 10,000 g for 30 min at 4°C. The collected supernatant, containing membrane and cytosolic fractions, was subjected to centrifugation in a JA30.50 rotor at 45,000 g for 90 min at 4°C. The resulting pellet containing membrane fraction was washed with a buffer composed by 5 mM Tris–HCl pH 7.4, 2 mM EDTA, 2 mM EGTA and 4 M urea, followed by another centrifugation cycle as above described. The washed membrane fraction was resuspended in a buffer composed by 25 mM Tris–HCl pH 7.4, 250 mM NaCl, 2 mM β-mercaptoethanol and 10% glycerol to reach a concentration of about 400 mg/ml. The membranes were homogenized with a potter and 3 mL aliquots were stored at −80°C before solubilization.

### Solubilization and Purification of hASCT2-6His

The purification of hASCT2-6His was performed starting from about 1.2 g of washed membranes (400 mg/ml) that were solubilized in a buffer composed by 25 mM Tris–HCl pH 7.4, 250 mM NaCl, 6 mM β-mercaptoethanol, 1 mM L-glutamine, 10% glycerol and 2% C_12_E_8_ (w/w). The solubilization was performed under rotatory stirring for 3 h at 4°C followed by centrifugation at 18,000 × *g* for 45 min. The supernatant was applied to 2 ml Ni-nitrilotriacetic acid (NTA) agarose resin pre-equilibrated with a buffer containing 20 mM Tris–HCl pH 7.4, 300 mM NaCl, 10% glycerol, 6 mM β-mercaptoethanol, 0.03% C_12_E_8_, 1 mM L-glutamine and 50 mM imidazole and incubated over-night, with gentle agitation, at 4°C. Then, the Ni-NTA resin was packed by gravity into a glass-column and washed with 30 ml of the same buffer above described. Elution of protein was, then, performed using 10 ml of a buffer containing 20 mM Tris–HCl pH 7.4, 300 mM NaCl, 10% glycerol, 6 mM β-mercaptoethanol, 0.03% C_12_E_8_, 1 mM L-glutamine and 500 mM imidazole. 2.5 ml of purified protein were pooled and desalted on a PD-10 column from which 3.5 ml were collected. The column was pre-equilibrated and eluted with a buffer composed by 20 mM Tris–HCl pH 7.4, 100 mM NaCl, 10% glycerol, 6 mM β-mercaptoethanol, 0.03% C_12_E_8_ and 1 mM L-glutamine.

### Inclusion of Cholesterol in Liposome Preparation

7.5 mg of cholesterol were added to 100 mg of egg yolk phospholipids and solubilized with 1 mL of chloroform obtaining a completely clear solution. After short incubation under rotatory stirring (30°C 5 min 1200 rpm) open tube is dried O.N. at room temperature. The dried lipid film was resuspended in 1 mL water (10% final concentration) and unilamellar liposomes were formed by two sonication cycles of 1 min (1 pulse ON and 1 pulse OFF, 40 W) with a Vibracell VCX-130 sonifier as previously suggested (Hanson et al., 2008).

### Reconstitution of the hASCT2-6His Into Liposomes

The purified hASCT2 was reconstituted by detergent removal in a batch-wise procedure. Mixed micelles of detergent, protein and phospholipids were incubated with 0.5 g Amberlite XAD-4 resin under rotatory stirring (1,200 rpm) at 23°C for 40 min as previously described (Scalise et al., 2019). The composition of the reconstitution was: 50 μl of the purified hASCT2 (5 μg protein), 2 mM EDTA, 220 μl of a mixture composed by 100 μl of 10% (w/v) egg yolk phospholipids (with or without included cholesterol as described in section 2.4) in the form of sonicated liposomes and 120 μl of 10% C_12_E_8_, 10 mM L-glutamine (or other amino acids as specified in the figure legend), 20 mM Hepes Tris pH 6.0 (or different pH as specified in the figure legend) and 10 mM NaCl (deriving from the purified protein and EDTA) in a final volume of 700 μl. All the operations were performed at room temperature.

### Transport Measurements

To remove the external compounds, 600 μL of proteoliposomes was passed through a sephadex G-75 column (0.7 cm diameter × 15 cm height) pre-equilibrated with a buffer composed by 20 mM Hepes Tris pH 6.0 and sucrose to balance the internal osmolarity. Uptake experiments were started in a 100 μl proteoliposomes sample by adding 50 μM [^3^H]glutamine or 500 μM [^3^H]glutamic acid (or other radiolabeled substrates as indicated in the figure legends) together with 50 mM Na-gluconate to, at 25°C. The transport reaction was stopped at the indicated times using 100 μM HgCl_2_. The control sample, blank, was prepared by adding the same inhibitor at time zero according to the inhibitor stop method (Palmieri and Klingenberg, 1979). At the end of the transport, 100 μL of proteoliposomes was passed through a sephadex G-75 column (0.6 cm diameter × 8 cm height) buffered with 50 mM NaCl to separate the external from the internal radioactivity. Then, proteoliposomes were eluted with 1 ml 50 mM NaCl and added with 3 ml of scintillation mixture, vortexed and counted. The experimental values were analyzed by subtracting to each sample the respective blank; the initial rate of transport was measured by stopping the reaction after 15 min, i.e., within the initial linear range of radiolabeled substrate uptake into the proteoliposomes.

### Spectrofluorometric Assays

The intraliposomal pH changes were monitored by measuring the fluorescence emission of pyranine included inside the proteoliposomes. Reconstitution mixture was performed as described in section 2.4 with some modifications: 25 μg purified protein was used for the reconstitution mixture and 0.1 mM pyranine at pH 7.0 was included in proteoliposomes. After reconstitution, 600 μL of proteoliposomes was passed through a sephadex G-75 column, pre-equilibrated with 20 mM Hepes Tris pH 7.0 and 10 mM sucrose, except where differently indicated. Then, 150 μL proteoliposomes were diluted in 3 ml of the same buffer containing 100 mM Na-gluconate, except where differently indicated. In the blank sample, 10 μM HgCl_2_ was added according to the stop inhibitor method above described. To start the transport assay, 5 mM glutamate buffered at pH 7.0, except where differently indicated, was added to induce glutamine_in_/glutamate_ex_ antiport; the uptake of protons was measured as a reduction of pyranine fluorescence included in proteoliposomes. The measurement was performed in the fluorescence spectrometer (LS55) from Perkin Elmer under rotatory stirring. The fluorescence was measured following time drive acquisition protocol with λ excitation = 450 nm and λ emission = 520 nm (slit 5/5) according to manufacturer instructions of pyranine. Calibration of the internal fluorescence changes vs. pH inside the proteoliposomes has been performed by reconstituting proteoliposomes with different pH buffers (from pH 6.0 to pH 7.5). Then, the fluorescence of internal pyranine was measured as function of the internal pH finding a linear correlation. The calibration was used to report the data of Figure 9B as nmol of protons transported inside proteoliposomes with glutamate and to calculate the stoichiometry of proton uptake vs. glutamate uptake.

### Sequence Alignment and Molecular Docking Approach

Multiple sequence alignment of SLC1 members was performed using Clustal Omega, after downloading amino acid sequences from UniProt. Docking analysis was performed using AutoDock Vina v.1.1.2 (Trott and Olson, 2010). The grid box was generated on the binding site, on the basis of glutamine coordination, and its size was set to 40 × 40 × 40 Å (x, y, and z) with spacing 0.375. Glutamate was downloaded from PubChem in sdf format. The ligand was prepared using LigPrep (Schrödinger, 2020a) within Schrödinger-Maestro v.12.4 (Schrödinger, 2020b). Default parameters were applied, except for the pH range since the ligand was prepared at two pH, 2.0 ± 0.5 and 7.0 ± 0.5, to have two different protonation states. Glutamate, with two different protonation states, was docked into refined ASCT2 (PDB ID: 6GCT, chain A). Lamarckian Genetic Algorithm was employed to search for the best conformation space of the ligand. Default parameters were used and 20 different conformations of glutamic acid were generated. The pose with the lowest binding energy conformation was chosen. Molecular visualization was performed with the UCSF Chimera v.1.14 software (Pettersen et al., 2004) (Resource for Biocomputing, Visualization, and Informatics, University of California, San Francisco, CA, United States).

### Cell Culture and Transport Assay in Intact Cells

HeLa cells were maintained in Dulbecco’s Modified Eagle Medium (DMEM) supplemented with 10% (v/v) fetal bovine serum (FBS), 1 mM sodium pyruvate and 4 mM glutamine. The cells were grown in a humidified incubator in a 5% CO2 atmosphere at 37°C. Cells for the transport assay were seeded into a 12 well dish up to 80% confluence. After 24 h, the medium was removed and the cells were washed twice with warm transport medium containing 20 mM Tris–HCl pH 7.4, 130 mM NaCl, 10 mM BCH, 10 mM MeAIB and 100 μM nimesulide. For efflux experiments, cells were allowed to take in 10 μM [^3^H]glutamine up to 10 min. Thus, uptake buffer was removed and cells were rinsed two times with 0.5 mL per well of ice-cold transport buffer containing 20 mM Tris–HCl pH 7.4; [^3^H]glutamine efflux was measured in 0.5 mL of transport buffer containing 130 mM NaCl and 20 mM Tris–HCl pH 7.0 or pH 6.0 as indicated in the figure legends in the presence of 10 mM different substrates. The efflux was measured within 1 min and cells from each well were solubilized in 500 μl of 1% TX-100 solution. Intracellular radioactivity was measured by adding 3 mL of Scintillation Cocktail to 400 μl of cell extract.

### Other Methods

To generate imposed membrane potential, valinomycin (0.75 μg/mg of phospholipid) was added to proteoliposomes prepared with intraliposomal 50 mM K-gluconate. Proteoliposomes were passed through sephadex-G75 and, then, treated with valinomycin for 30 sec before transport measurement to allow potassium diffusion downhill its concentration gradient. To generate an artificial pH gradient (ΔpH), nigericin (0.15 μg/mg phospholipid) was added to proteoliposomes in the presence of an inwardly directed K^+^ gradient. Proteoliposomes were passed through sephadex-G75 and, then, treated with nigericin for 30 sec before transport measurement to allow potassium/proton exchange. For DEPC treatment, proteoliposomes were incubated after passage through sephadex G-75 column with 5 mM DEPC solution for 5 min under rotatory stirring (1,200 rpm) at 23°C prior transport measurement. Stock solutions of valinomycin, nigericin and DEPC in ethanol were prepared daily, and intermediate dilutions of this reagent into buffer were prepared immediately before use. The amount of purified recombinant hASCT2 WT was estimated from Coomassie blue-stained 12% SDS–PAGE gels by using the ChemiDoc imaging system equipped with Quantity One software (Bio-Rad) as previously described (Giancaspero et al., 2015).

### Data Analysis

Results are expressed as means ± SD. GraFit 5.0.13 software was used to calculate kinetic parameters, to derive per cent of residual activity values in inhibition assays and to measure transport rate by first-order rate equation. The statistical significance of experimental data was assessed by Student’s test for *p* < 0.05 as specified in the figure legends.

## Results

### Effect of Glutamate on the Glutamine Transport by hASCT2 Reconstituted in Proteoliposomes

**FIGURE 1:**
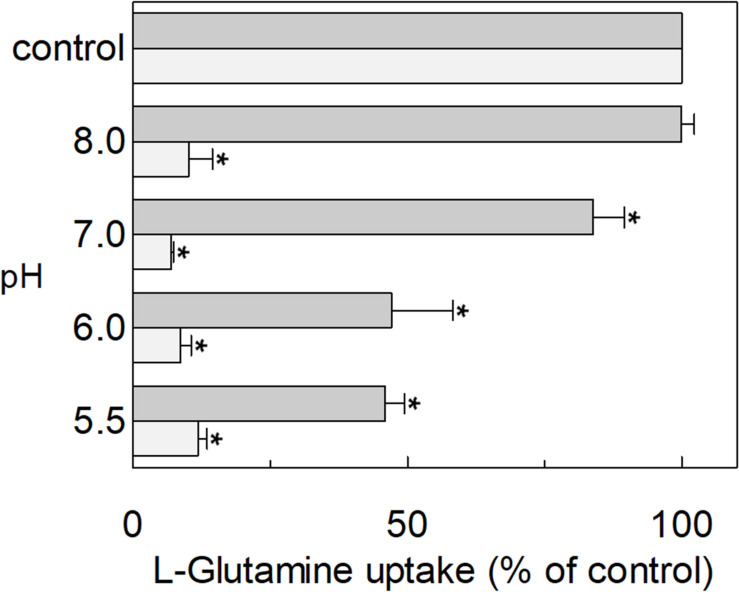
Effect of glutamate and alanine on the glutamine transport by hASCT2 reconstituted in proteoliposomes. Purified hASCT2 was reconstituted in proteoliposomes prepared at the indicated pH and containing 10 mM glutamine, as described in materials and methods. The transport assay was started by adding 50 μM [^3^H]glutamine, buffered at the indicated pH, in the presence of 50 mM Na-gluconate and 1 mM glutamate (gray bar) or 1 mM alanine (light gray bar). The transport was measured in 15 min according to the stop inhibitor method. The transport was indicated as % [^3^H]glutamine uptake with respect to the condition with no addition (control). Results are means ± S.D. from three independent experiments. *Significantly different from the control as estimated by Student’s *t*-test (*P* < 0.05).

**FIGURE 2:**
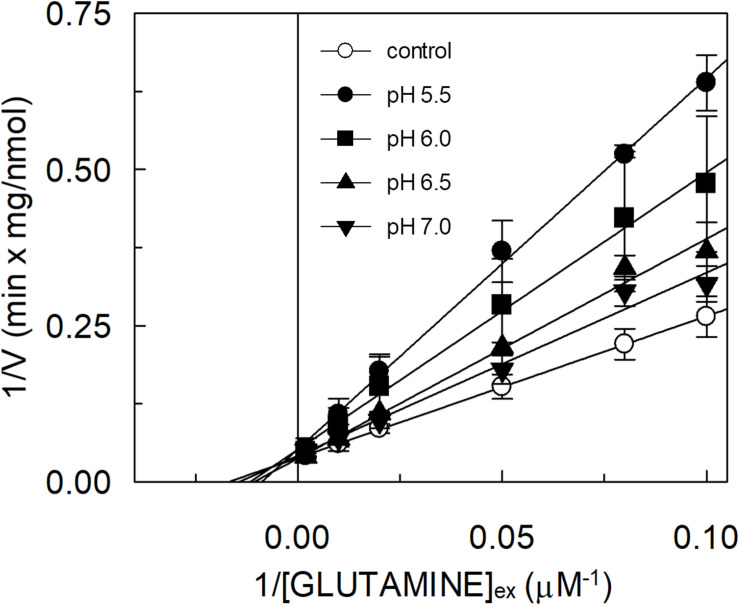
Effect of glutamate on the kinetics of glutamine transport by hASCT2 reconstituted in proteoliposomes. Purified hASCT2 was reconstituted in proteoliposomes prepared at the indicated pH and containing 10 mM glutamine, as described in materials and methods. The transport was started by adding indicated concentrations of [^3^H]glutamine and 50 mM Na-gluconate to proteoliposomes in the presence of 5 mM glutamate buffered at different pH: pH 5.5 (●), pH 6.0(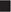), pH 6.5 (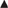), pH 7.0 (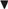). The control condition (○) represents a mean of [^3^H]glutamine measurements at the different pH (5.5, 6.0, 6.5, and 7.0) being Km for glutamine not affected by pH variations. The transport was measured in 15 min according to the stop inhibitor method. Data were plotted according to linear Lineweaver–Burk equation as reciprocal transport rate vs. reciprocal glutamine concentration. Results are means ± SD from three independent experiments.

The hASCT2 was reconstituted in proteoliposomes and the transport was assayed as Na^+^-[^3^H]glutamine_ex_/glutamine_in_ antiport. The effect of glutamate added to the proteoliposomes was tested on the transport function. As shown in Figure 1, glutamate inhibits the glutamine antiport in a pH-dependent fashion; the extent of inhibition varied substantially being maximal, around 50%, at acidic pH and very low or null at pH 7.0 and pH 8.0, respectively. The pH-dependent effect was specific for glutamate: indeed, when alanine was added to the proteoliposomes at the same concentration of glutamate, the inhibition ranged from 80 to 90%, being nearly pH-independent. As expected, the extent of inhibition by alanine was stronger than that by glutamate, given the higher affinity of ASCT2 toward alanine (Zander et al., 2013; Scalise et al., 2014). To characterize the inhibition, kinetic experiments were conducted (Figure 2). The dependence of the transport rate on glutamine concentration was measured in the presence of 5 mM glutamate at different pH values from pH 5.5 to pH 7.0. The pattern depicted in Figure 2 is typical of competitive inhibition, suggesting that glutamate binds to the glutamine binding site and, hence, it could be a potential substrate of the transporter.

### Transport of Glutamate by hASCT2 Reconstituted in Proteoliposomes

**FIGURE 3:**
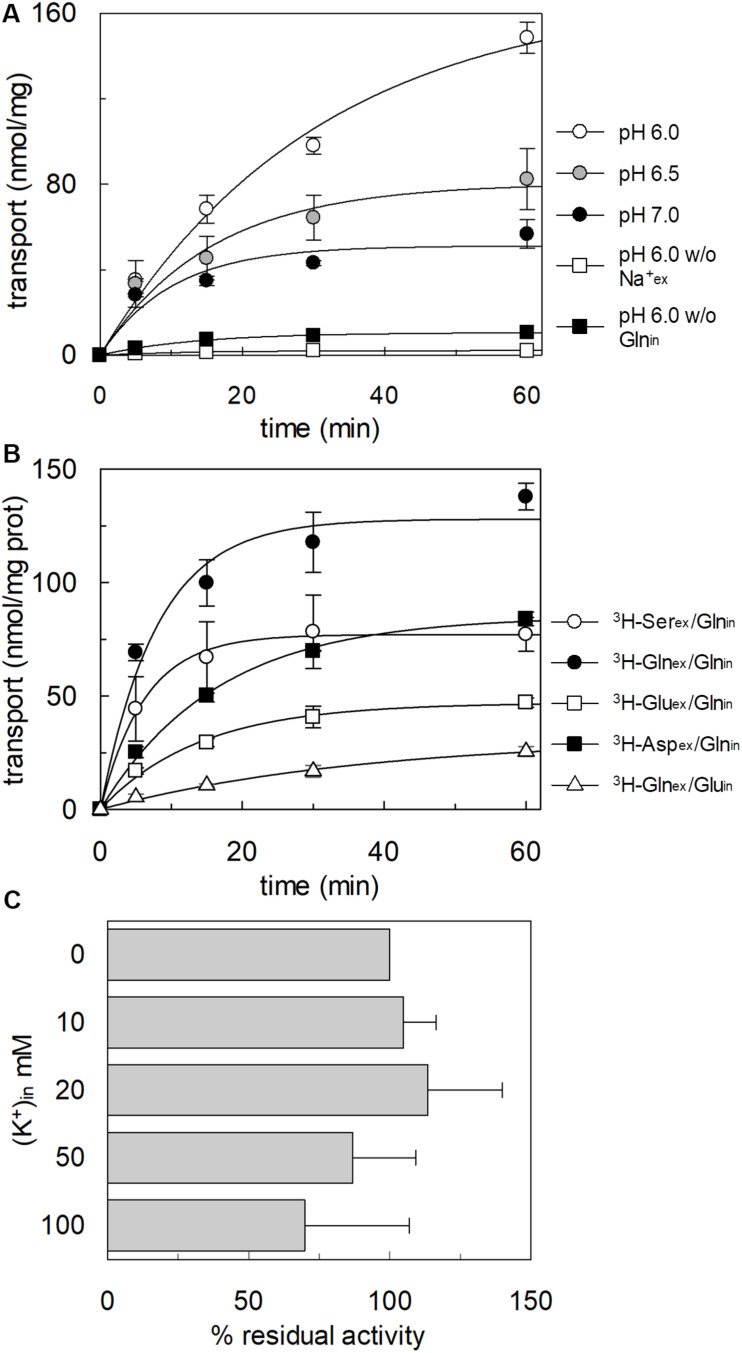
Functional characterization of glutamate transport by hASCT2 reconstituted in proteoliposomes. Purified hASCT2 was reconstituted in proteoliposomes as described in materials and methods. In **(A)**, Uptake of [^3^H]glutamate. The transport was started by adding 500 μM of [^3^H]glutamate in the absence (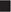) or the presence (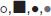) of 50 mM Na-gluconate to proteoliposomes containing (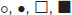) or not (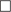) 10 mM glutamine. Proteoliposomes and radiolabeled substrate were prepared at pH 6 (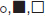), pH 6.5 (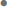), or pH 7(●). In **(B)**, Uptake of [^3^H]serine, [^3^H]asparagine, [^3^H]glutamine, and [^3^H]glutamate. The transport was started by adding 50 μM [^3^H]serine_ex_ (○) or [^3^H]asparagine (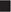) or [^3^H]glutamine (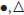) or 500 μM [^3^H] glutamate (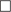) together with 50 mM Na-gluconate to proteoliposomes containing 10 mM glutamine (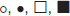) or 10 mM glutamate (△). In **(A,B)**, the transport was measured at the indicated times according to the stop inhibitor method. Data were plotted according to the first-order rate equation. In **(C)**, Dependence on internal K^+^ of the [^3^H]glutamate transport. Indicated concentrations of K-gluconate were added together with 10 mM glutamine in the intraliposomal compartment. The transport assay was started by adding 500 μM of [^3^H]glutamate together with 50 mM Na-gluconate. The transport was measured in 60 min according to the stop inhibitor method. The values were expressed as percent residual activity with respect to the control condition that is no K^+^ in (0 mM K^+^). Results are means ± SD from three independent experiments. No significant difference from the control was observed as estimated by Student’s *t*-test (*P* < 0.05).

**FIGURE 4:**
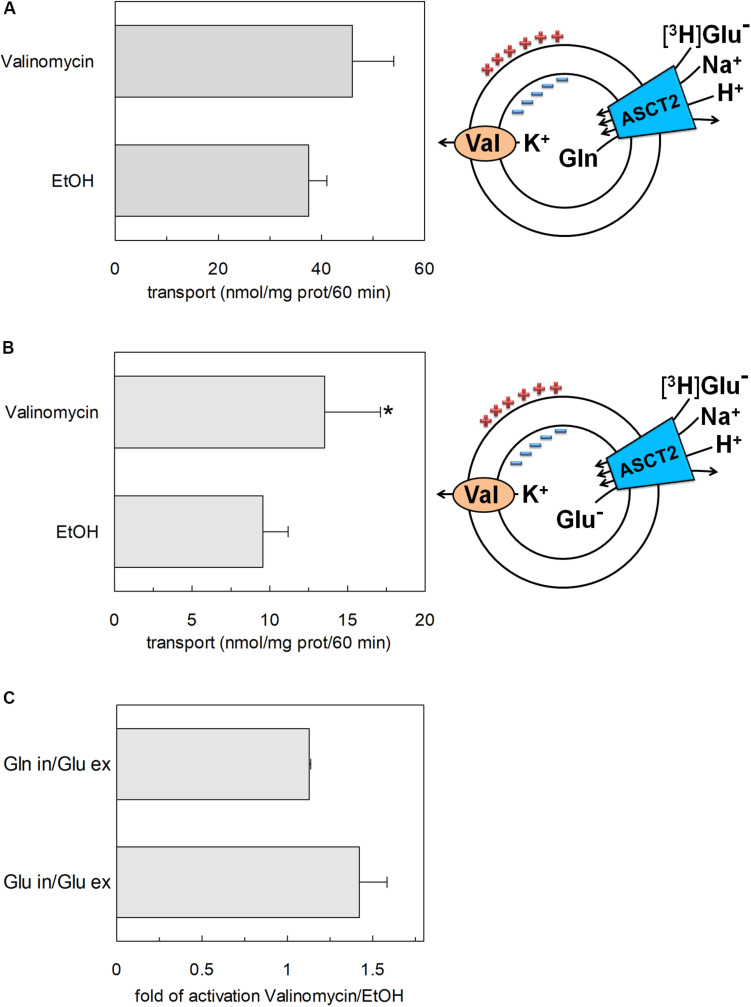
Effect of imposed membrane potential on glutamate transport by hASCT2 reconstituted in proteoliposomes. Purified hASCT2 was reconstituted in proteoliposomes as described in materials and methods. After sephadex-G75 chromatography, proteoliposomes were treated with valinomycin (0.75 μg/mg phospholipid) to impose the artificial membrane potential. The vehicle of valinomycin, ethanol (EtOH) was used as control of valinomycin treatment. The transport assay was started adding 500 μM of [^3^H]glutamate together with 50 mM Na-gluconate to proteoliposomes containing 10 mM glutamine **(A)** or glutamate **(B)** and 50 mM K-gluconate. The transport was measured in 60 min according to the stop inhibitor method. The transport rate was expressed as nmol/mg in 60 min. In **(A,B)** sketch of the experimental set up (proteoliposomes); Val: valinomycin. In **(C)**, data from **(A,B)** were used to calculate the fold of activation valinomycin/ethanol. Results are means ± SD from four independent experiments. *Significantly different from the control sample (EtOH) as estimated by Student’s *t*-test (*P* < 0.05).

To confirm that hASCT2 could mediate the glutamate transport, the [^3^H]glutamate uptake in proteoliposomes containing internal glutamine, in the presence of external sodium, was measured. As shown by the figure, the reconstituted transporter is indeed able to mediate the uptake of [^3^H]glutamate as a function of time (Figure 3A). In line with the results from Figure 2, the uptake of [^3^H]glutamate is dependent on pH with an optimum at pH 6.0. According to the peculiar three-substrate reaction of hASCT2, the transport of [^3^H]glutamate requires both internal glutamine and external sodium. Indeed, the uptake was not measurable in the absence of the internal substrate or external sodium. To compare the efficiency of the Na^+^[^3^H]glutamate_ex_/glutamine_in_ antiport with that of other neutral amino acids, the antiport of [^3^H]serine or [^3^H]asparagine in exchange for internal glutamine was measured (Figure 3B). The transport rate of [^3^H]glutamate_ex_/glutamine_in_ antiport is only four or two times lower than that of [^3^H]serine_ex_/glutamine_in_ or [^3^H]asparagine_ex_/glutamine_in_, respectively. Interestingly, the reverse reaction, i.e., [^3^H]glutamine_ex_/glutamate_in_ was the least stimulated suggesting that glutamate is preferentially transported from the external to the internal side of cells, in line with the feature of the glutamine/glutamate cycle (Leke and Schousboe, 2016). The activity of glutamate transporters belonging to the SLC1 family is dependent on intracellular potassium; then, increasing concentrations of intraliposomal (intracellular) potassium were tested on the [^3^H]glutamate uptake via hASCT2 (Figure 3C). None or negligible effect was measured on hASCT2 transport activity. Moreover, the electric nature of the Na^+^ [^3^H]glutamate_ex_/glutamine_in_ antiport was investigated imposing a K^+^ diffusion membrane potential in the presence of valinomycin, as previously described for the Na^+^ [^3^H]glutamine_ex_/glutamine_in_ antiport (Pingitore et al., 2013; Scalise et al., 2014). This triggered a slight stimulation of the Na^+^[^3^H]glutamate_ex_/glutamine_in_ transport (Figure 4A). In line with the electrical nature of the vectorial reaction, the homoexchange Na^+^[^3^H]glutamate_ex_/glutamate_in_ (Figure 4B) is more stimulated than the heteroexchange Na^+^[^3^H]glutamate_ex_/glutamine_in_ (Figure 4C). However, the homoexchange (Glu_ex_/Glu_in_) is less efficient than the heteroexchange (Glu_ex_/Gln_in_) in line with the results obtained from the experiments reported in Figure 3B.

### Kinetics of Glutamate Transport by hASCT2 Reconstituted in Proteoliposomes

**FIGURE 5:**
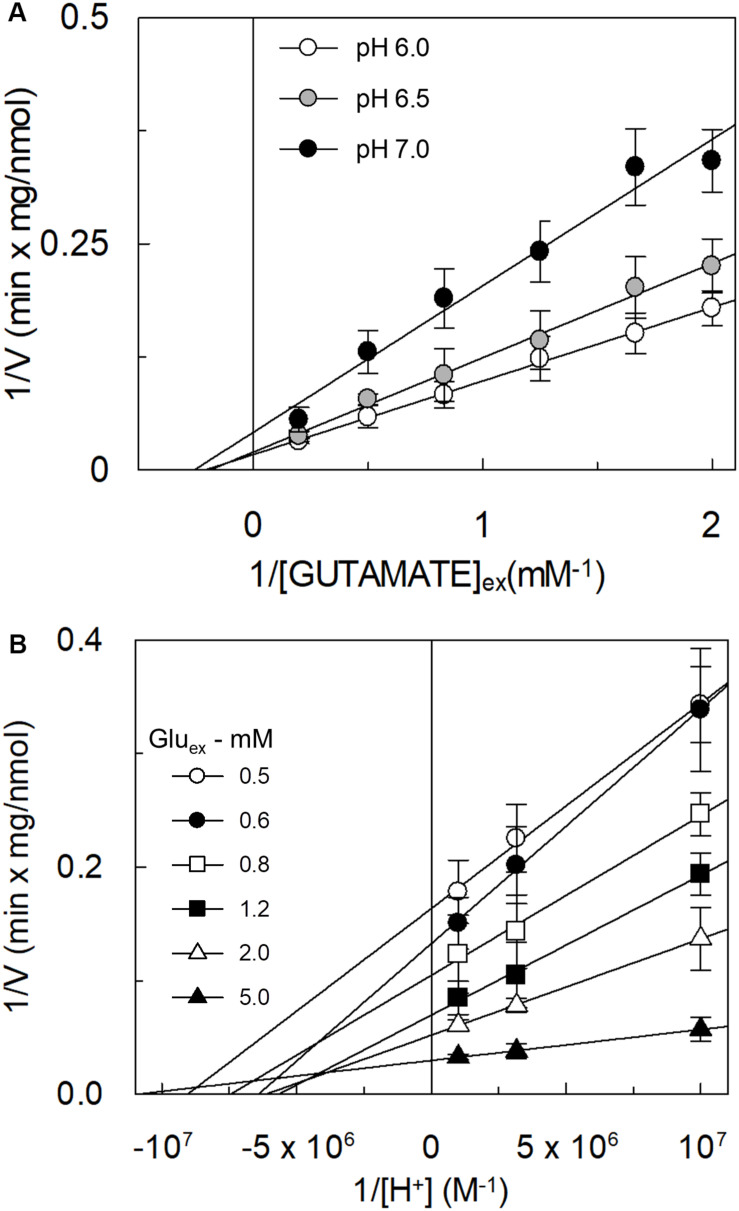
Effect of pH on the kinetics of glutamate transport by hASCT2 reconstituted in proteoliposomes. Purified hASCT2 was reconstituted in proteoliposomes prepared at the indicated pH and containing 10 mM glutamine, as described in materials and methods. In **(A)**, the transport was started by adding indicated concentrations of [^3^H]glutamate and 50 mM Na-gluconate to proteoliposomes buffered at different pH: pH 6.0 (○), pH 6.5 (●), and pH 7 (●). The transport was measured in 15 min according to the stop inhibitor method. Data were plotted according to linear Lineweaver–Burk equation as reciprocal transport rate vs. reciprocal glutamate concentration. In **(B)**, data from Figure 5A were replotted according to Lineweaver-Burk equation as reciprocal transport rate vs. reciprocal proton concentration, performed at different glutamate concentration: 0.5 mM (○), 0.6 mM (●), 0.8 mM (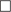), 1.2 mM (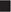), 2 mM (△), and 5 mM (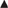). The derived pKa values were as follows: 7.0 ± 0.2, 6.8 ± 0.2, 6.9 ± 0.2, 6.8 ± 0.2, 6.8 ± 0.2, 7.0 ± 0.2 at 0.5, 0.6, 0.8, 1.2, 2, and 5 mM glutamate, respectively. Results are means ± SD from five independent experiments.

The kinetic analysis of the [^3^H]glutamate_ex_/glutamine_in_ antiport was performed as the dependence of the transport rate on glutamate concentration at different pH conditions (Figure 5A). The collected data showed that the Km was virtually not affected by the pH, while the Vmax doubled at pH 6.0 with respect to pH 7.0. Km values in the millimolar range were obtained at the different pH values: 4.8 ± 1.9 mM, 5.3 ± 1.4 mM and 3.9 ± 1.5 mM at pH 6.0, pH 6.5, and pH 7.0, respectively. The data reported in Figure 5A were re-plotted against proton concentration to evaluate the affinity of the hASCT2 toward proton at different glutamate concentrations, reported in Figure 5B. Interestingly, the Km toward proton was only slightly affected by the glutamate concentrations in a range from 0.5 to 5 mM, being around a proton concentration corresponding to pH 7.0. On the contrary, Vmax increased by increasing glutamate concentration. It is worthy of note that the range of pH used in these experiments is above the pKa of the side chain carboxylic group of glutamate; in these conditions, the majority (from 98.3% at pH 6.0 to 99.8% at pH 7.0) of glutamate is in a dissociated form, suggesting that the proton should not be bound to glutamate during the transport process.

### Involvement of Proton in the Transport of Glutamate by hASCT2 in Proteoliposomes

**FIGURE 6:**
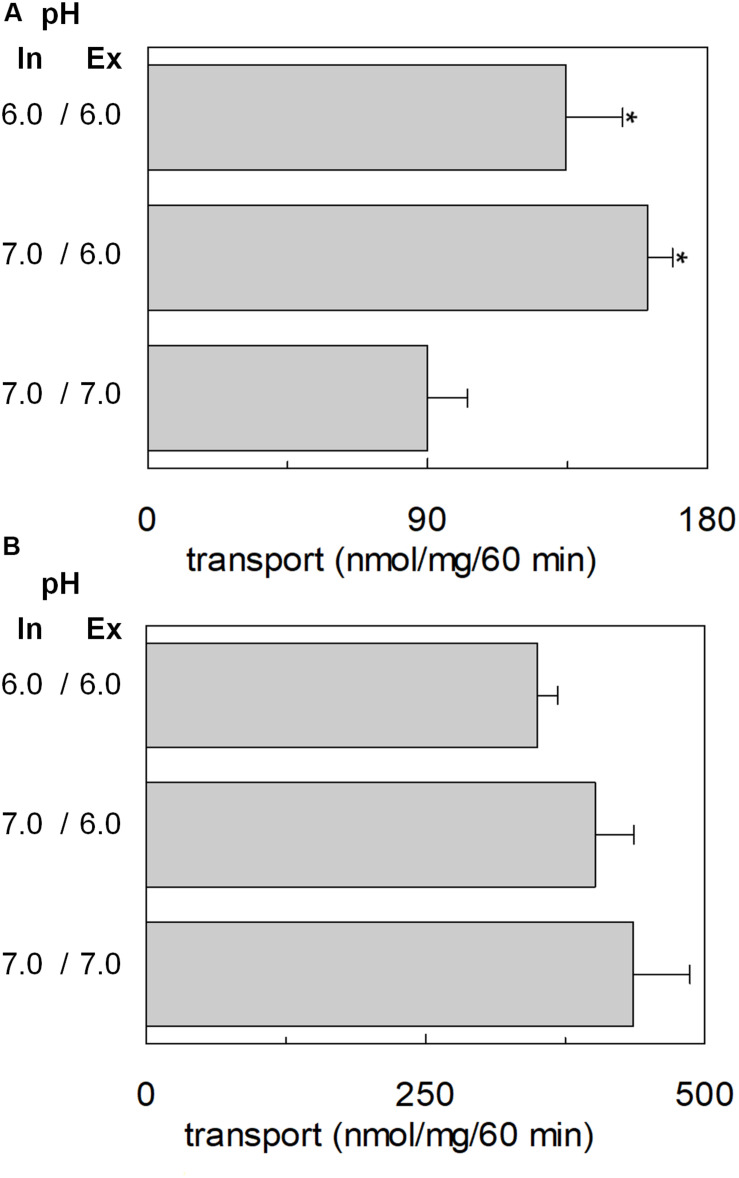
Effect of ΔpH on glutamate transport by hASCT2 reconstituted in proteoliposomes. Purified hASCT2 was reconstituted in proteoliposomes as described in materials and methods, buffered at the indicated intraliposomal pH (in). The transport assay was started by adding 50 mM Na-gluconate and 500 μM of [^3^H]glutamate **(A)** or 50 μM of [^3^H]glutamine **(B)** prepared at the indicated pH (ex). The transport was measured in 60 min according to the stop inhibitor method. The transport rate was expressed as nmol/mg in 60 min. Results are means ± SD from three independent experiments. *Significantly different from the control sample (pH 7.0_in_/7.0_ex_) as estimated by Student’s *t*-test (*P* < 0.05).

**FIGURE 7:**
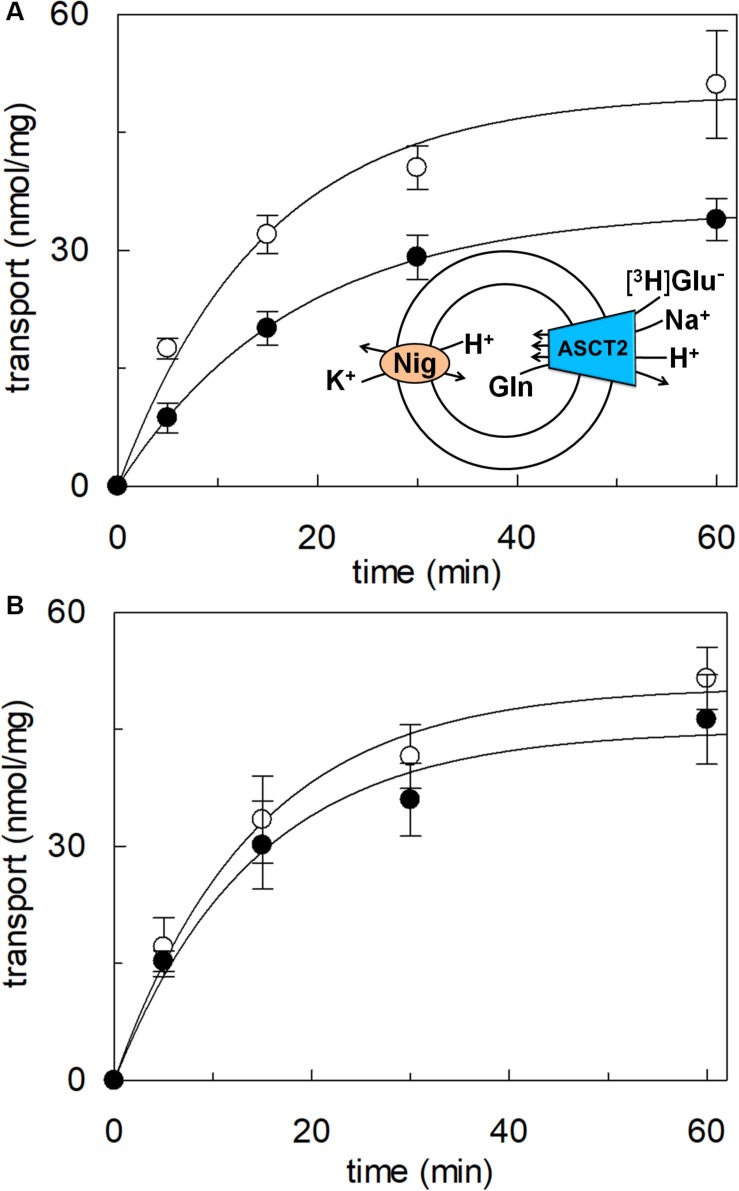
Effect of imposed membrane proton gradient on glutamate transport by hASCT2 reconstituted in proteoliposomes. Purified hASCT2 was reconstituted in proteoliposomes as described in materials and methods. After sephadex-G75 chromatography, proteoliposomes were treated with nigericin (0.12 μg/mg phospholipid) (○) to impose the artificial membrane proton gradient. The vehicle of nigericin, ethanol (●) was used as control of nigericin treatment. The transport assay was started adding 500 μM of [^3^H]glutamate together with 20 mM Na-gluconate and 30 mM K-gluconate to proteoliposomes containing 10 mM glutamine **(A)**. In **(B)**, the transport was performed in the absence of external K-gluconate, as control. In the figure, sketch of the experimental set up (proteoliposomes); Nig: nigericin. The transport was measured at the indicated times according to the stop inhibitor method. Data were plotted according to the first-order rate equation. Results are means ± SD from four independent experiments.

The transport of glutamate via the high-affinity glutamate transporters of the SLC1 family occurs together with the movement of a proton across the plasma membrane (Zerangue and Kavanaugh, 1996). Therefore, the possible involvement of proton in the transport of glutamate via hASCT2 was investigated. According to the results described in Figure 5, two hypotheses could explain the collected data: (i) the proton is transported along a different pathway than that of glutamate; (ii) the proton binds to a transporter site, facilitating the glutamate transport, but is not transported itself. To discriminate between the two possibilities, we employed different approaches. At first, the [^3^H]glutamate_ex_/glutamine_in_ antiport was measured in the presence of a ΔpH (Figure 6). In particular, the transport of [^3^H]glutamate in proteoliposomes was stimulated by a pH gradient pH 6.0_ex_/pH 7.0_in_ if compared to the conditions with no gradient, i.e., pH 7.0_ex_/pH 7.0_in_ or pH 6.0_ex_/pH 6.0_in_ (Figure 6A). This feature was specific for the glutamate transport; indeed, when the same experiment was conducted using [^3^H]glutamine, the transport in the presence of the pH gradient was less active than the control condition at pH 7.0_ex_/pH 7.0_in_, i.e., the optimal transport condition for the glutamine antiport (Figure 6B). To confirm the observed effect on the Na^+^[^3^H]glutamate_ex_/glutamine_in_, a pH gradient was generated by using nigericin, an ionophore able to mediate the exchange of K^+^ with H^+^ (sketch in Figure 7A). In this condition, an inwardly directed pH gradient was generated and an increased [^3^H]glutamate uptake was observed in comparison to the control, i.e., no nigericin (Figure 7A). The extent of activation is about 75% at the initial transport rate, as derived from the time-course analysis. In good agreement with this data, when the same experiment was conducted without K^+^ in the extraliposomal compartment, no stimulation was observed (Figure 7B).

### Transport of Proton by hASCT2 in Proteoliposomes

**FIGURE 8:**
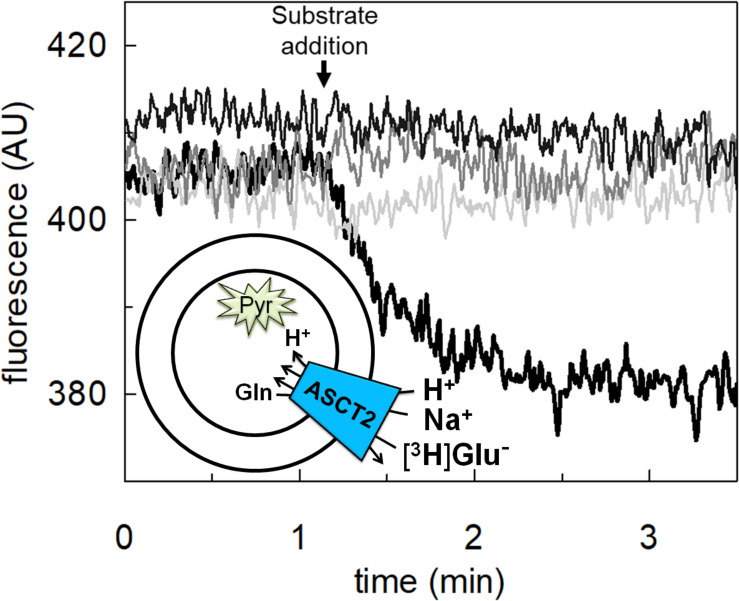
Transport of proton by hASCT2 reconstituted in proteoliposomes. Purified hASCT2 was reconstituted in proteoliposomes containing 10 mM glutamine and 0.1 mM pyranine as described in materials and methods. The fluorescence measurement was started by adding 150 μL proteoliposomes to the transport buffer (up to 3 mL final volume) composed by Hepes Tris 20 mM pH 7 and 100 mM Na-gluconate. After ∼1 min, as indicated by the arrow, 5 mM glutamate (thick black line) or 1 mM glutamine (gray line) was added to the sample and fluorescence change was recorded. As a control, the same measurement was performed in the absence of Na-gluconate (black line); in the blank sample, 10 μM HgCl_2_ was added at time 0 to the transport buffer (light-gray line). In the figure, sketch of the experimental set up (proteoliposomes); Pyr: pyranine. The fluorescence signal is indicated as arbitrary units (AU). Results are representative of three independent experiments.

**FIGURE 9:**
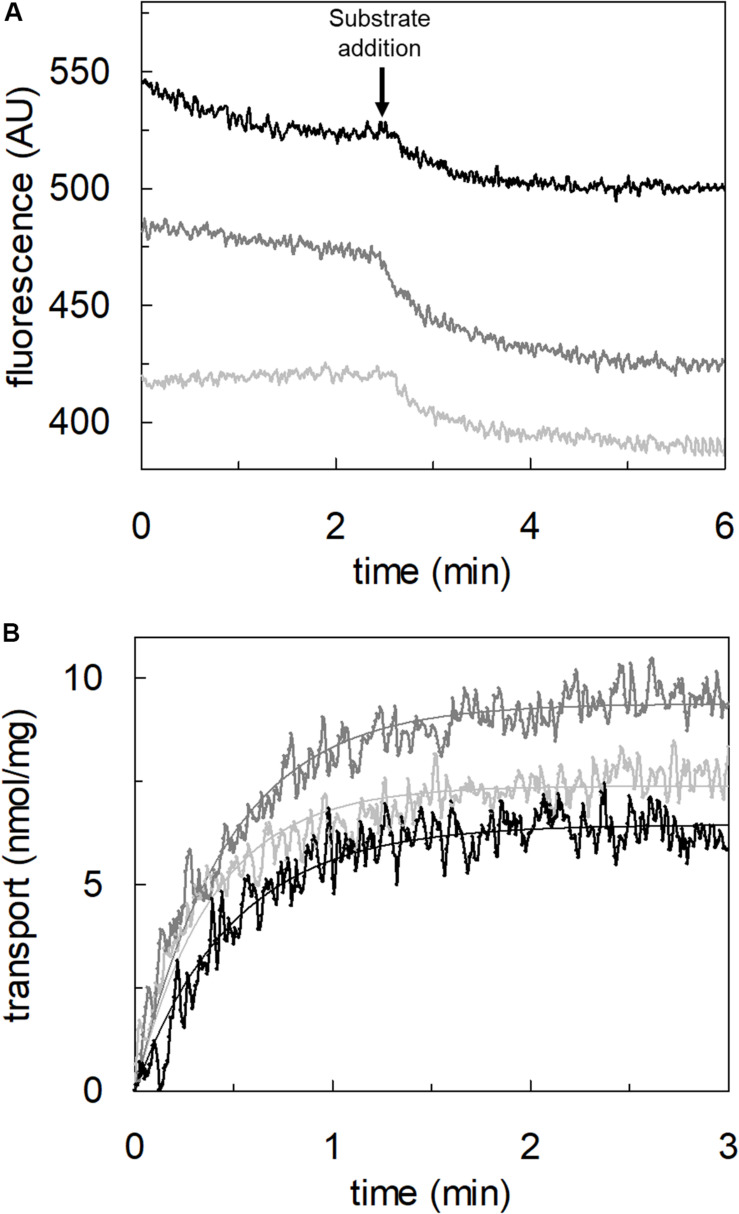
Effect of pH on proton transport by hASCT2 reconstituted in proteoliposomes. Purified hASCT2 was reconstituted in proteoliposomes buffered at pH 7.0, containing 10 mM glutamine and 0.1 mM pyranine, as described in materials and methods. In **(A)**, the fluorescence measurement was started by adding 150 μL proteoliposomes to the transport buffer (up to 3 mL final volume) composed by 100 mM Na-gluconate and Hepes Tris 20 mM at pH 6.0 (light gray) or pH 6.5 (gray) or pH 7.0 (black). After ∼2 min, as indicated by the arrow, 5 mM buffered glutamate was added to the sample and fluorescence change was recorded. The fluorescence signal is indicated as arbitrary units (AU). In **(B)**, data from **(A)** were converted in nmol of transported proton measured as nmol/mg; the calculation was performed after calibrating the fluorescence signal assuming a linear relationship between fluorescence decrease and proton uptake (Gibrat and Grignon, 2003). Data were plotted according to a first-order rate equation. Results are representative of three independent experiments.

**FIGURE 10:**
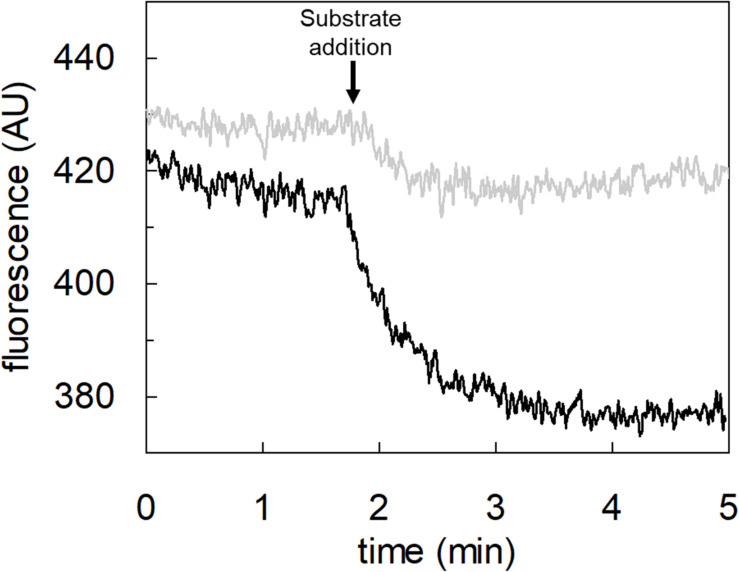
Effect of DEPC on the proton transport by hASCT2 reconstituted in proteoliposomes. Purified hASCT2 was reconstituted in proteoliposomes buffered at pH 7.0, containing 10 mM glutamine and 0.1 mM pyranine, as described in materials and methods. After reconstitution, proteoliposomes were incubated with 10 mM DEPC (gray line) or ethanol (black line) for 5 min at 23°C and, then were subjected to sephadex-G75 chromatography. The fluorescence measurement was started by adding 150 μL proteoliposomes to transport buffer (up to 3 mL final volume) composed by 100 mM Na-gluconate and Hepes Tris 20 mM at pH 7.0. After ∼2 min as indicated by the arrow, 5 mM glutamate was added to the sample and fluorescence was recorded. The fluorescence signal is indicated as arbitrary units (AU). Results are representative of three independent experiments.

To achieve the final proof of proton movement across the membrane, an alternative strategy was employed based on spectrofluorometric measurement using the pH-sensitive dye pyranine whose fluorescence emission increases with the pH (Giuliano and Gillies, 1987; Indiveri et al., 1994). Therefore, pyranine was included in the intraliposomal compartment (see Materials and methods) and the uptake of protons was measured in the presence of glutamate or glutamine externally added (Figure 8). As shown in the figure, the addition of glutamate caused a time-dependent decrease of the fluorescence, in line with the uptake of protons coupled to that of glutamate. As a control, the addition of glutamine did not induce any fluorescence changes, i.e., protons were not taken up during the glutamine/glutamine antiport reaction. According to the sodium dependence of glutamate transport, proton movement was not detected when sodium was not included in the transport buffer. Finally, the proton uptake, i.e., fluorescence decrease, was abolished by the addition of the inhibitor, HgCl_2_ (Figure 8). The dependence on pH was also evaluated in the spectrofluorometric assay (Figure 9); the decrease of fluorescence, i.e., the increase of proton concentration in the intraliposomal compartment, is indeed pH-dependent (Figure 9A). The maximal fluorescence decrease is observed at pH 6.5. To quantify the extent of proton transport, the data from Figure 9A was used to calculate the nmol of proton taken up in proteoliposomes at different pH values (Figure 9B). The uptake of protons in terms of specific transport, i.e., nmol/mg protein taken up, was in the same order of magnitude of that measured for glutamate under similar experimental conditions (Figure 5A), being in favor of a 1:1 stoichiometry for H^+^:Glu^–^ transport. The hASCT2 harbors in its primary structure four histidine residues; therefore, we argued that at least one of them could be involved in the proton transport. Then, DEPC was employed for spectrofluorometric measurements performed at pH 6.5 (Figure 10). Interestingly, the decrease of fluorescence, i.e., the proton flux toward the intraliposomal compartment, was reduced in the presence of 10 mM DEPC.

### Docking of Glutamate to hASCT2

**FIGURE 11:**
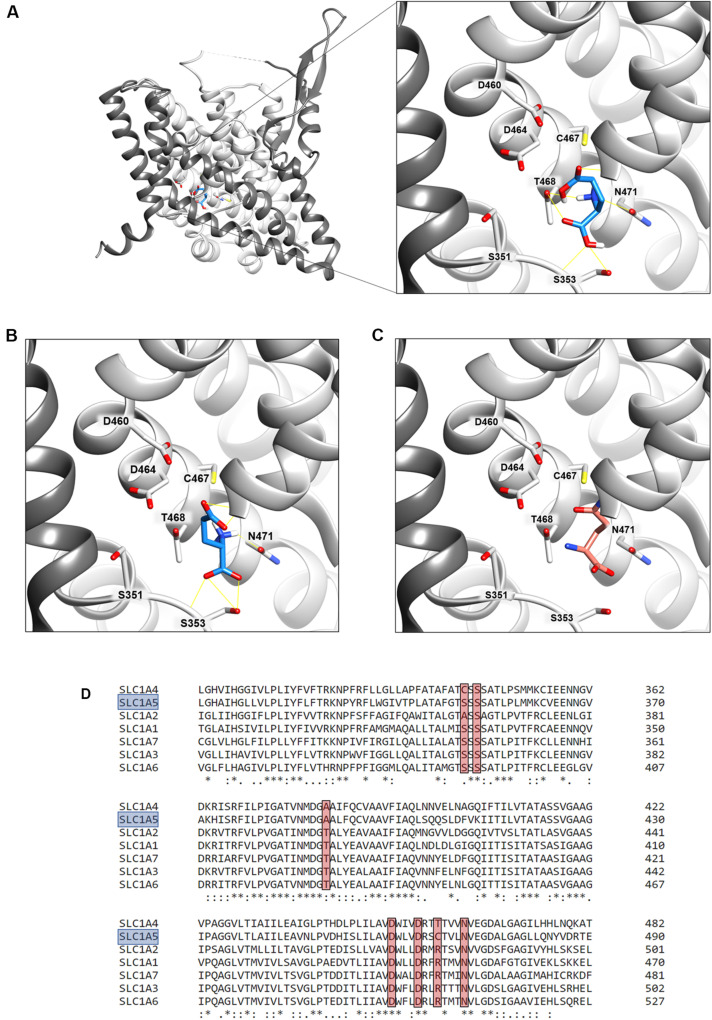
Sequence alignment and docking analysis of the hASCT2 binding site. In **(A,B)**, Molecular docking of glutamic acid in the binding site of ASCT2. The crystal structure of ASCT2 in the inward open conformation has been used (PDB ID: 6GCT). To simplify the visualization, only the Chain A without Cholesterol was represented as light gray ribbon, with the scaffold domain in dim gray, using Chimera v.1.14. In **(A)**, a zoom of protonated glutamate docking in the binding site. The ligand was represented as dodger blue stick and amino acids belonging to the binding site as a stick and labeled using Chimera v.1.14. Glutamate in the protonated state was prepared at a pH range of 2.0 ± 0.5 using Schrödinger-Maestro v.12.4 and docking was performed using AutoDock Vina v.1.1.2 as described in materials and methods. This pose has a docking score of −6.0 and it makes hydrogen bonds with G435, T468, N471, and S353. In **(B)**, a zoom of deprotonated glutamate docking in the binding site. The ligand was represented as dodger blue sticks and amino acids belonging to the binding site are represented as a stick and labeled using Chimera v.1.14. Glutamate in the deprotonated state was prepared at a pH range of 7.0 ± 0.5 using Schrödinger-Maestro v.12.4 and docking was performed using AutoDock Vina v.1.1.2 as described in Methods. The best pose has a docking score of −6.1 and it makes interaction with G430, G434, G435, N471, and S353. In **(C)** visualization of the glutamine binding site. Glutamine is represented as salmon sticks and the amino acids belonging to the binding site are represented as a stick and labeled using Chimera v.1.14. In **(D)**, Multiple sequence alignment of SLC1 members. To simplify, the multiple alignment is represented from L311 to E490 referring to SLC1A5 (boxed in light blue). Residues belonging to the binding site (A390, S351, S353, D460, D464, C467, N471 in SLC1A5) are highlighted with a red box.

The binding site for neutral amino acids on hASCT2 was previously identified by a site-directed mutagenesis approach (Scalise et al., 2018a) and then confirmed by the recently obtained 3D structure (Garaeva et al., 2018, 2019; Yu et al., 2019). The competitive inhibition data reported in Figure 2, suggested that also glutamate binds to the substrate-binding site of hASCT2. To further support this biochemical finding, we performed docking analysis on the available 3D structure of hASCT2 in the inward open conformation (PDB 6GCT) (Figures 11A,B). Glutamate, both in the protonated (Figure 11A) and deprotonated (Figure 11B) state, positioned similarly to glutamine (Figure 11C), docked as a control (Figure 11C). Interestingly, docked glutamate did not directly interact with Cys467 but with the residues of the hASCT2 substrate-binding site conserved among the SLC1 family members (Figure 11D).

### Transport of Aspartate by hASCT2 Reconstituted in Proteoliposomes

**FIGURE 12:**
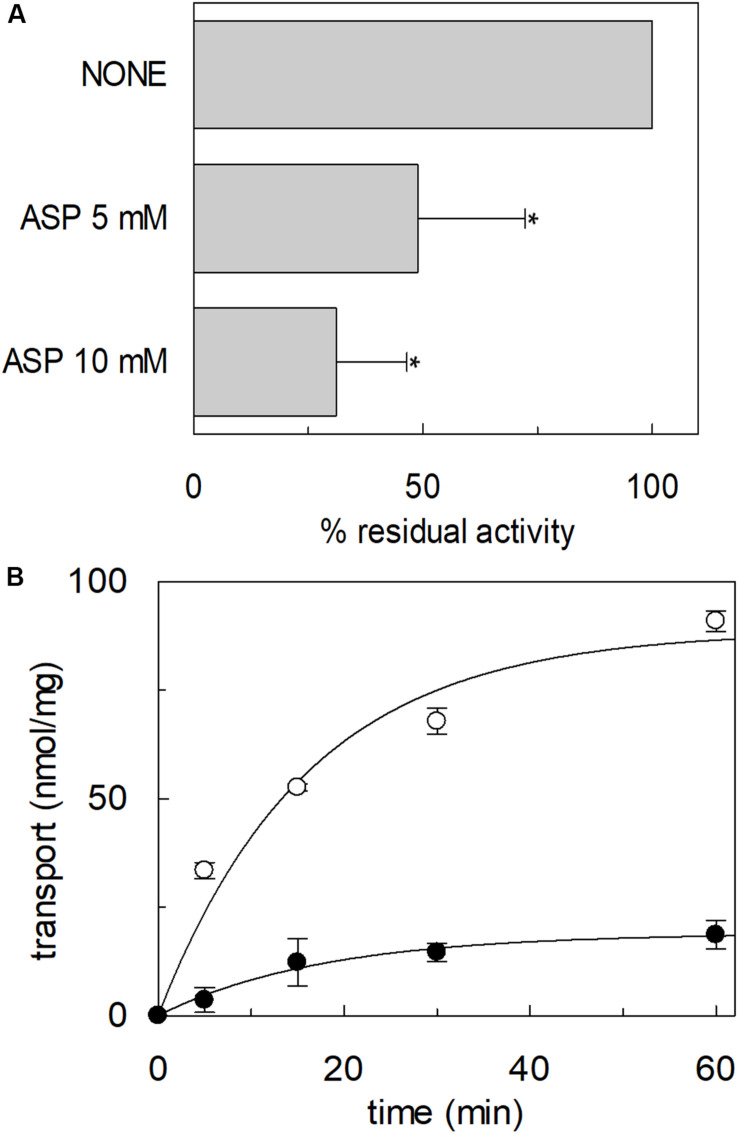
Interaction of hASCT2 reconstituted in proteoliposomes with aspartate. Purified hASCT2 was reconstituted in proteoliposomes containing 10 mM glutamine as described in materials and methods. In **(A)**, inhibition by aspartate of the glutamate transport by ASCT2 reconstituted in proteoliposomes. The transport assay was started by adding 500 μM [^3^H]glutamate together with 50 mM Na-gluconate to proteoliposomes in the presence of indicated concentrations of aspartate. The transport was measured in 60 min according to the stop inhibitor method. The transport was indicated as % residual activity with respect to the condition with no external addition (none). Results are means ± SD from four independent experiments. *Significantly different from the control sample (none) as estimated by Student’s *t*-test (*P* < 0.05). In **(B)**, uptake of glutamate and aspartate by ASCT2 in proteoliposomes. The transport assay was started by adding 500 μM [^3^H]glutamate (○) or 500 μM [^3^H]aspartate (●) together with 50 mM Na-gluconate to proteoliposomes. The transport was stopped at the indicated times according to the stop inhibitor method. Data were plotted with the first-order rate equation. Results are means ± SD from three independent experiments.

The high-affinity glutamate transporters of the SLC1 family can mediate the flux of aspartate as well. Therefore, the inhibition of the [^3^H]glutamate transport by aspartate added to the extraliposomal compartment was evaluated; indeed, aspartate inhibited the hASCT2 mediated glutamate transport (Figure 12A). Then, we evaluated the direct transport of [^3^H]aspartate via hASCT2 reconstituted in proteoliposomes; interestingly, as it is shown in Figure 12B, the transport of aspartate was lower than that of glutamate in line with the lack of complete inhibition of glutamate transport (Figure 12A).

### Glutamine Efflux Induced by Glutamate via hASCT2 in Intact Cells and Proteoliposomes

**FIGURE 13:**
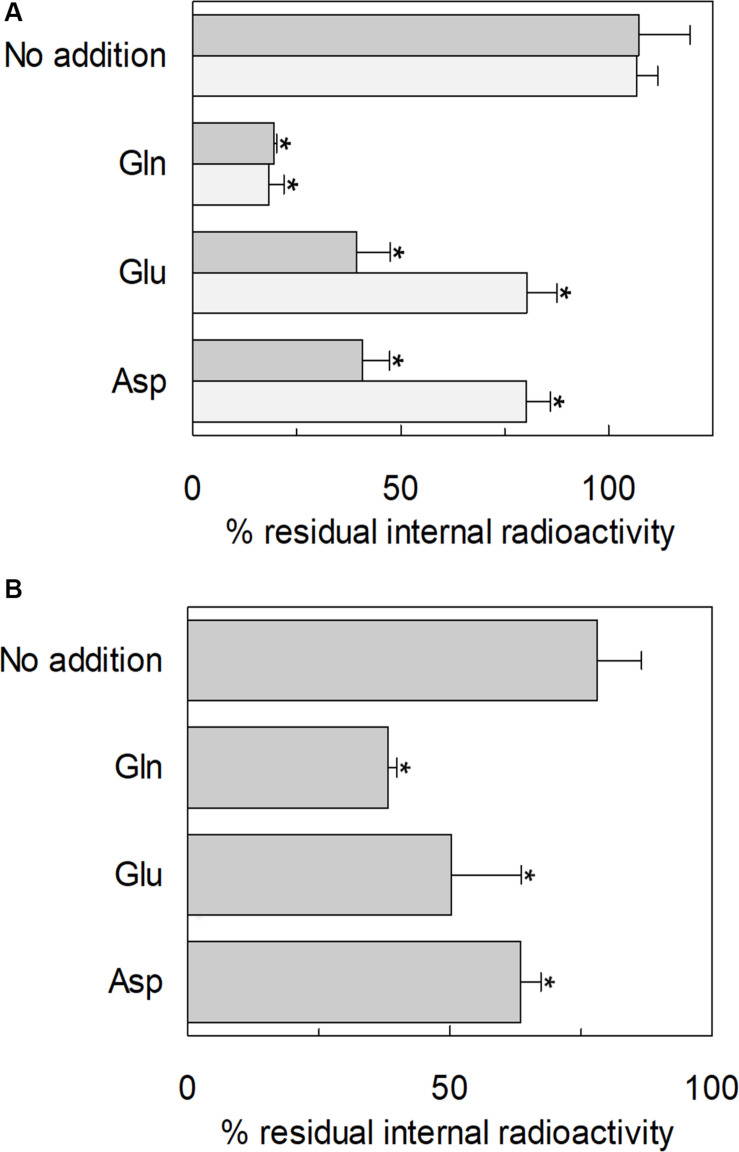
Efflux of glutamine through hASCT2. In **(A)** [^3^H]glutamine efflux from proteoliposomes. Purified hASCT2 was reconstituted in proteoliposomes containing 10 mM glutamine as described in materials and methods. The accumulation of radiolabeled glutamine was started by adding 50 μM [^3^H]glutamine in the presence of 50 mM Na-gluconate. After 60 min uptake, the [^3^H]glutamine efflux was started in different conditions: no addition (control), the addition of 10 mM of glutamate or aspartate or 1 mM glutamine to the transport buffer in the presence of 50 mM Na-gluconate. The efflux was measured in 60 min using substrates buffered at pH 7.0 (light gray) or pH 6.0 (dark gray). In **(B)**, Efflux of [^3^H]glutamine from HeLa intact cells. Cells were cultured as described in materials and methods; uptake was performed in 10 min adding 10 μM [^3^H]glutamine in the presence of 20 mM Tris–HCl pH 7.0 and 130 mM NaCl as described in materials and methods. The efflux was measured in 1 min in the presence of different conditions: no addition (control), the addition of 10 mM of glutamate or aspartate or 1 mM glutamine to the transport buffer constituted by 20 mM Tris–HCl pH 6.0 and 130 mM NaCl. Results are means ± SD from four independent experiments. *Significantly different from the control sample (no addition) as estimated by Student’s *t*-test (*P* < 0.05).

The ability of glutamate to induce efflux of glutamine was investigated in both proteoliposomes and intact cells (Figure 13). In line with the data from Figure 3, glutamate was able to induce the efflux of [^3^H]glutamine from preloaded proteoliposomes (Figure 13A). As shown by the figure, externally added glutamate caused a reduction of intraliposomal radioactivity of about 65% at pH 6.0. As controls, glutamine or buffer alone was externally added: 85% or 0% efflux was detected, respectively. The data confirmed that hASCT2 could mediate the antiport of glutamate_ex_/[^3^H]glutamine_in_, even though at a lower extent with respect to glutamine_ex_/[^3^H]glutamine_in_. In line with the ability of hASCT2 to mediate also aspartate transport, a similar extent of efflux was observed adding aspartate in the place of glutamate. In good agreement with the uptake of radiolabeled glutamate, the described phenomenon is also pH-dependent; indeed, the addition of glutamate or aspartate buffered at pH 7.0, caused a lower efflux compared to that measured at pH 6.0. As a control, in the case of glutamine, nearly no difference was observed at the different pH values. A similar experiment was performed in intact HeLa cells, which are known to express ASCT2 and have been previously used for studying ASCT2 transport (Torres-Zamorano et al., 1998; Scalise et al., 2014, 2019). Also in this case, glutamate and aspartate evoked a glutamine efflux of about 50% and 25%, respectively, in comparison to the control. Glutamine, as expected, induced a greater efflux, reaching about 60%. Collectively, the data from intact cells and proteoliposomes confirmed the ability of glutamate to induce glutamine efflux in line with the glutamine/glutamate cycle (Leke and Schousboe, 2016).

## Discussion

### Transport of Glutamate via hASCT2

In this work, different assays have been employed to shed light on the substrate specificity of hASCT2; this represents a vintage but very relevant issue in the understanding of hASCT2 biology in both physiological and pathological conditions with potential outcomes in pharmacology. In line with very early but disregarded observations on mice and rat ASCT2 (Utsunomiya-Tate et al., 1996; Broer et al., 1999; Oppedisano et al., 2007), the capacity of the hASCT2 to mediate a Na^+^ and H^+^ dependent glutamate_ex_/glutamine_in_ antiport emerged. The rate of this reaction is comparable to the well-assessed antiport of neutral amino acids (Figure 3B). As a matter of fact, the substrate-binding site of wild type hASCT2 can accept glutamate and, at a lower extent, aspartate (Figure 12). The critical variation, with respect to the conventional hASCT2 transport mode, is the requirement for acidic pH that suggests the involvement of protons in the transport mechanism, reminiscent of the other glutamate transporters belonging to the SLC1 family, i.e., the EAAT members. Based on the kinetic data on WT hASCT2, it can be assumed that the transported form of glutamate is the anionic one (Figure 5); this is in line with the observation that the Km values toward glutamate do not change when varying the pH of the transport assay, reflecting the concentration of glutamate in the anionic form. It is then expected that the proton *flux* occurs through an independent transport path, even though at this stage, we cannot completely exclude that the proton might be transported together with glutamate.

### Transport of Proton via hASCT2

The availability of recombinant hASCT2 reconstituted in proteoliposomes allowed to directly measure the proton movement by fluorescence changes induced by the glutamate transport. This approach represents a methodological novelty in the study of the transporter; the advancement was made feasible thanks to the improvement of the functional protein fraction following the discovery that cholesterol facilitates the formation of the trimeric functional assembly of the protein (Garaeva et al., 2018, 2019; Scalise et al., 2019). These achievements resulted in a substantial increase in the active space of proteoliposomes and, hence, of the volume available for the fluorescence detection by pyranine (Indiveri et al., 1994). The results collected by the spectrofluorometric assays correlated well with the transport assay of glutamate by radioactivity. The stoichiometry of transport is in favor of an overall Na^+^ dependent transport reaction of 1H^+^:1Glu^–^ in exchange for internal glutamine. From the kinetic data reported in Figure 5, a further biochemical conclusion can be drawn: the pH dependence of glutamate transport, that is maximal at pH 6.0, is in line with the Km toward proton calculated as a proton concentration corresponding to pH 7.0 (Figure 5B). Indeed, when measuring glutamate transport at pH 6.0, the proton concentration is 10 times above the Km, i.e., close to saturation, correlating well with the doubling of the Vmax of glutamate transport from pH 7.0 to pH 6.0. In line with the proposed charge movement, valinomycin imposed membrane potential positively affects the glutamate transport via hASCT2. In good agreement with the hypothesis that glutamate is transported in the anionic form, the stimulation by the imposed membrane potential is amplified when the glutamate^–^_ex_/glutamate^–^_in_ antiport is measured: the homologous glutamate/glutamate antiport, *per se*, is indeed electroneutral with consequent net inward movement of two positive charges deriving from sodium and proton. In the case of glutamate^–^_ex_/glutamine_in_ exchange, the negative charge of glutamate compensates one of the co-transported positive charges. It has to be stressed that the charge movement accounts only partially in the overall driving force of transport, that mainly derives by the antiport component and the sodium gradient (Scalise et al., 2014) similarly to what previously observed for the rat ASCT2 (Zander et al., 2013). The electrogenic nature of the glutamate_ex_/glutamine_in_ antiport is in agreement with the role of allosteric regulation by internal Na^+^, similar to that described in the case of the neutral amino acid antiport (Scalise et al., 2014). Noteworthy, when measuring the glutamine_ex_/glutamate_in_ antiport, the transport activity was lower if compared to the *vice versa* reaction (Figure 3B); besides the affinity issue, this can be explained by the electrogenicity of the hASCT2 mediated transport. Indeed, the exit of a net negative charge due to glutamate^–^ movement, would create a charge unbalance positive inside which counteracts the movement of Na^+^ and H^+^, from the external environment.

### Transport of Acidic Amino Acids Within the SLC1 Family

Interestingly and differently from the high-affinity glutamate transporters of the SLC1 family, the glutamate transport via hASCT2 does not require internal potassium. This is in good agreement with data collected in the pre-3D structure era, in which a residue of glutamate of EAAT2 (previously known as GLT-1), namely E404, was identified as responsible for the EAAT-specific potassium stimulation; indeed, ASCTs does not harbor a glutamate residue in the corresponding position but a neutral glutamine residue (Kavanaugh et al., 1997; Grewer et al., 2003). Still in agreement with our data, the EAAT2-E404Q mutant is not sensitive to intracellular potassium (Grewer et al., 2003). The layout of hASCT2 binding site able to coordinate both neutral and negatively charged amino acids is a distinctive trait of hASCT2 with respect to the other members of SLC1 family (Figure 11). Over the years, several mutations have been generated on ASCT1 and EAATs to switch substrate specificity of these proteins. In the case of hASCT2, the presence of C467 seems to increase the range of acceptable substrates depending on the pH. This may find a possible explanation in the size of the cysteine side chain. Indeed, in old papers, it was already highlighted that cysteine, smaller than arginine of EAATs and smaller than threonine of ASCT1, creates a larger niche to accommodate substrates (Bendahan et al., 2000). Later on, the 3D structure of hASCT2 showed the presence of a narrow space, between scaffold and transport domain, that forms a larger space directed to the intracellular side; this feature is peculiar of hASCT2 (Freidman et al., 2020). Furthermore, recent work showed that, even if lysine is not recognized as a substrate of hASCT2, small amino acids, modified to have basic side chains, can be translocated by hASCT2 and rASCT2 (Ndaru et al., 2020). Also in this case, the basic amino acid substrates do not interact with C467. The interaction of protons with the high-affinity glutamate transporters has been investigated in the very early studies conducted on EAAT2. The key residues H326, and a stretch constituted by D398, E404 and D470, were identified by site-directed mutagenesis. Interestingly, these amino acids are conserved in all the EAATs (Pines et al., 1995) and a similar mechanism was described for the proton dependent lac permease (Sahin-Toth and Kaback, 2001). Interestingly, in hASCT2, three out of the four mentioned residues are conserved, suggesting that they could be part of the proton path in the protein. Investigations are in the course for identifying the transport path of protons and glutamate in hASCT2.

### Glutamate and Aspartate Transport in Physiological and Pathological Conditions

Besides the mere biochemical feasibility of the exchange of external glutamate or aspartate with internal glutamine, this vectorial reaction is plausible to take place also in the actual physiological context, as suggested by the experiments conducted on intact cells (Figure 13B). Indeed, the presence of ASCT2 in the placenta may account for the glutamate/glutamine cycle between placenta and fetus to sustain the metabolic requirement (Vaughn et al., 1995; Torres-Zamorano et al., 1998; Day et al., 2013). A similar role may be suggested for ASCT2 in the glutamate/glutamine cycle between neurons and astrocytes to sustain the activity of EAATs, i.e., the major players in the glutamate reuptake from the synaptic cleft to avoid glutamate excitotoxicity (Leke and Schousboe, 2016). It has to be noted that in synaptic cleft the transient concentration of glutamate may reach 100-200 mM (Meldrum, 2000; Gras et al., 2006), that is much higher than the Km of ASCT2 for glutamate. However, given the major role played by EAATs and the relatively low expression of ASCT2 in the adult brain, it can be speculated that the role of ASCT2 in the reuptake of glutamate in the brain may become relevant in pathological conditions characterized by transient pH changes, such as in hypoxia (Deitmer and Rose, 1996). Besides the transport of glutamate, a physiological explanation of recognizing negatively charged amino acids by hASCT2, relies on the transport of aspartate across the BBB, as previously suggested in mice to maintain the homeostasis of excitatory amino acids in the brain (Tetsuka et al., 2003). A glutamate/glutamine cycle has been proposed also in activated macrophages in CNS following HIV infection, as a neuroprotective mechanism (Gras et al., 2006). Recently, a glutamate/glutamine cycle has been suggested to occur between cancer and stromal cells to sustain the metabolic changes (Lyssiotis and Kimmelman, 2017; Bott et al., 2019). Interestingly, a pH-dependent glutamate uptake was described in the intestine, where luminal pH is acidic. This glutamate transport was attribute to the ASC transporter by competitive inhibition studies (Munck and Munck, 1999; Munck et al., 2000). From the described scenario, it can be speculated that the substrate specificity of ASCT2 may change according to the tissues in which the protein is expressed following also developmental stage and/or post-translational modifications that may refine the preference toward one or more amino acids. Furthermore, the possibility of mediating the uptake of glutamate and aspartate further enlarges the significance of ASCT2 overexpression in the context of cancer metabolic rewiring; as an example, aspartate is required for nucleotide biosynthesis and glutamate is required for redox balance in cancer initiation and progression (Lieu et al., 2020). Taken together, the novelties on substrate specificity here presented further points out that ASCT2 is one of the key players for the metabolism of highly proliferative cells. Furthermore, the identification of other substrates, rather than neutral amino acids, may have also a relevance in providing new clues for pharmacological research. Indeed, ASCT2 is considered an eminent druggable target and the design of drugs able to specifically interact with this protein is subjected to its improved biochemical characterization.

## Data Availability Statement

The raw data supporting the conclusions of this article will be made available by the authors, without undue reservation, to any qualified researcher.

## Author Contributions

MS and CI conceived, designed the experiments, and analyzed the data. MS and TM performed the proteoliposome functional assays. JC and FR performed the docking analysis. LP and TM prepared the yeast constructs and optimized yeast cell growth. GP performed the yeast cell growth for protein over-expression and purification. MS, TM, and CI wrote the manuscript. CI supervised the entire work. All authors contributed to the article and approved the submitted version.

## Conflict of Interest

The authors declare that the research was conducted in the absence of any commercial or financial relationships that could be construed as a potential conflict of interest.

## Abbreviations

BBB: Blood Brain Barrier
BMGY: Buffered Glycerol-complex Medium
BMMY: Buffered Methanol-complex Medium
C_12_E_8_: Octaethylene glycol monododecyl ether
CNS: Central Nervous System
CryoEM: CryoElectron Microscopy
DEPC: diethyl pyrocarbonate
DMEM: Dulbecco’s Modified Eagle Medium
DTE: DiThioErythritol
SLC: SoLute Carrier
YPDS: Yeast Extract Peptone Dextrose Sorbitol.
